# Notes

**Published:** 1899-08-19

**Authors:** 


### NOTES.

A fever hospital has been established at Hebburn, containing typhoid and scarlet fever wards and the usual offices, at a cost of £4,000.

Under the will of the late Mr. Thomas Lockwood, of Bilton House, Harrogate, the Bradford Infirmary and the Harrogate Royal Bath Hospital will receive £2,000 each; and the Bradford Eye and Ear Hospital, the Huddersfield Infirmary, and the Harrogate Cottage Hospital £1,000 each.

A new dispensary has been added to the Portsmouth Workhouse Infirmary. The building consists of the dispensary, surgery, steward's office, store, and disinfecting-room. The infirmary has also undergone other improvements, the whole of the sanitary arrangements having been renewed, the hot-water service remodelled, and the whole of the interior of the institution repainted and redecorated.

A new wing has been opened by Sir Stafford Northcote at the Tiverton Infirmary which will serve to commemorate the Queen's Jubilee by the town. Not only is the building new, but it also embodies a new movement on the part of infirmary authorities, as the extra accommodation provided is to be devoted to the reception of paying patients. Two wards, each with two beds, have been provided for the purpose. Besides these the building contains nurses' quarters, a waiting-room, operating-room, bath-room, and the heating apparatus for the entire infirmary. The cost of the addition has been £1,550.

The danger of wooden hospitals was again illustrated by a fire which broke out last week in the administrative block of the Sheffield Infectious Diseases Hospital at Lodge Moor. This institution consists of long ranges of wooden one-storey buildings, which were erected about 10 years ago for the accommodation of small-pox patients, and at present it has 130 inmates. Fortunately the flames were confined to the block in which they originated.

A complaint has been put forward at the Cardiff Infirmary that the amount spent upon viands for the resident medical staff is too large. This is estimated at 25s. per head, and a special committee has been appointed to go into the matter. Indignation was expressed by some of the doctors present that any effort at economy in the direction of meals for the staff should have been suggested. Perhaps these gentlemen would be surprised at the excellent fare that can with good management be supplied at little over half the mentioned cost.

